# Transcriptome profiling of anthocyanin biosynthesis in the peel of ‘Granny Smith’ apples (*Malus domestica*) after bag removal

**DOI:** 10.1186/s12864-019-5730-1

**Published:** 2019-05-09

**Authors:** Changqing Ma, Bowen Liang, Bo Chang, Jiuying Yan, Li Liu, Ying Wang, Yazhou Yang, Zhengyang Zhao

**Affiliations:** 1 State Key Laboratory of Crop Stress Biology for Arid Areas, College of Horticulture, Northwest A & F University, Yangling, 712100 Shaanxi China; 2 Shaanxi Research Center of Apple Engineering and Technology, Yangling, 712100 Shaanxi China

**Keywords:** Anthocyanin content, Apple bagging, Differentially expressed genes, Granny Smith cultivar, RNA sequencing, *MdMYB1*

## Abstract

**Background:**

Bagging is commonly used to enhance red pigmentation and thereby improve fruit quality of apples (*Malus domestica*). The green-skinned apple cultivar ‘Granny Smith’ develops red pigmentation after bagging removal, but the signal transduction pathways mediating light-induced anthocyanin accumulation in apple peel are yet to be defined. The aim of this study was to identify the mechanisms underpinning red pigmentation in ‘Granny Smith’ after bag removal based on transcriptome sequencing.

**Results:**

The anthocyanin content in apple peel increased considerably after bag removal, while only trace amounts of anthocyanins were present in the peel of unbagged and bagged fruits. RNA sequencing identified 18,152 differentially expressed genes (DEGs) among unbagged, bagged, and bag-removed fruits at 0, 4, and 10 days after bag removal. The DEGs were implicated in light signal perception and transduction, plant hormone signal transduction, and antioxidant systems. Weighted gene co-expression network analysis of DEGs generated a module of 23 genes highly correlated with anthocyanin content. The deletion of − 2026 to − 1870 bp and − 1062 to − 964 bp regions of the *MdMYB1* (LOC103444202) promoter induced a significant decrease in glucuronidase activity and anthocyanin accumulation in apple peel.

**Conclusions:**

Bagging treatment can induce red pigmentation in ‘Granny Smith’ via altering the expression patterns of genes involved in crucial signal transduction and biochemical metabolic pathways. The − 2026 to − 1870 bp and − 1062 to − 964 bp regions of the *MdMYB1* promoter are essential for *MdMYB1*-mediated regulation of anthocyanin accumulation in the ‘Granny Smith’ apple cultivar. The findings presented here provide insight into the mechanisms of coloration in the peel of ‘Granny Smith’ and other non-red apple cultivars.

**Electronic supplementary material:**

The online version of this article (10.1186/s12864-019-5730-1) contains supplementary material, which is available to authorized users.

## Background

Apple (*Malus domestica*) is a highly valued and widely cultivated fruit crop around the world. From a consumer’s perspective, apples with red peel are preferred to other colors in terms of their appearance and nutritional value [1]. The red color of apple peel is due to the presence of anthocyanins [2], a natural source of antioxidants with high nutritional value and potential health benefits [3, 4].

Anthocyanins are biosynthesized through the flavonoid pathway that involves many structural and regulatory genes [5, 6]. The structural genes associated with anthocyanin biosynthesis in apple have been characterized, including phenylalanine ammonia lyase (PAL), chalcone synthase (CHS), 4-coumarate coenzyme A ligase (4CL), chalcone isomerase (CHI), dihydroflavonol 4-reductase (DFR), flavanone 3-hydroxylase (F3H), anthocyanidin synthase/leucoanthocyanidin dioxygenase (ANS), and UDP-glucose:flavonoid 3-O-glucosyltransferase (UFGT) [7, 8]. All these structural genes are regulated by a conserved MYB-bHLH-WD40/WDR (MBW) regulatory complex [9]. Transcriptional levels of *MYB* are correlated with anthocyanin biosynthesis in red-skinned apple cultivars, and in red regions of apple peel [10]. By regulating the levels of structural gene transcripts, methylation of the apple *MdMYB1* promoter affects the formation of red pigments in apple peel [11, 12]. A single-nucleotide polymorphism in the *MdMYB1* promoter causes abnormalities of anthocyanin biosynthesis [13].

Anthocyanin accumulation in plant can be regulated by light and plant hormones [14, 15]. Under light conditions, plant photoreceptors perceive and transduce light signals to regulate anthocyanin biosynthesis [16]. The classical photoreceptors include phytochromes (PHYs), cryptochromes (CRYs), and phototropins (PHOTs) that perceive light signals ranging from ultraviolet (UV)-A to far-red [17]. Exposure to UV-B irradiation promotes anthocyanin biosynthesis, resulting in binding and activation of promoter regions of *MdMYB* genes in apple peel [18]. UVR8 is a UV-B photoreceptor that plays vital roles in UV-B induction of flavonoid biosynthesis and plant defense against UV-B [19–21]. Downstream of the photoreceptors, CONSTITUTIVE PHOTOMORPHOGENIC 1 (COP1) [17], LONG HYPOCOTYL 5 (HY5) [16], suppressor of phyA (SPA) [22], DE-ETIOLATED (DET) [23], and PHYTOCHROME KINASE SUBSTRATE 1 (PKS1) [24] also participate in light-induced plant development. In addition, plant hormones regulate the expression of anthocyanin biosynthetic genes in a light-dependent manner [25]. For example, jasmonic acid modulates anthocyanin biosynthetic genes expression toward the end of the pathway, while cytokinins induce the expression of both early and late anthocyanin biosynthetic genes in *Arabidopsis* [26].

Anthocyanins play a photoprotective role under certain stress conditions, such as high light exposure [27]. The photoprotective function of anthocyanins may be mainly attributed to direct scavenging of reactive oxygen species (ROS) [28, 29]. Various enzymatic antioxidant systems are present in cells, including superoxide dismutase (SOD), catalase (CAT), peroxidase (POD), ascorbate peroxidase (APX), glutathione reductase (GR), monodehydroascorbate reductase (MDHAR), glutathione peroxidase (GPX), and glutathione-S-transferase (GST), that work in concert to prevent uncontrolled oxidation [30–32]. In apples, the relationship between anthocyanin biosynthesis and antioxidant systems is dependent on light conditions. Unlike the non-red cultivar ‘Golden Delicious’, antioxidant systems in the red cultivar ‘Red Delicious’ are initially upregulated by anthocyanins during sunlight exposure, but at higher anthocyanin concentrations downregulation can occur [33]. However, whether these enzymatic antioxidant systems are also involved in red pigmentation in non-red apple cultivars after bag removal have not been well documented.

The ‘Granny Smith’ is a green-skinned apple cultivar, but fruits can turn cardinal red after bag removal, as practiced in the Loess Plateau region of China to improve apple quality [34]. Recently, several studies have attempted to explain this coloration phenomenon in ‘Granny Smith’. The major anthocyanin pigment present in ‘Granny Smith’ peel is cyanidin 3-galactoside [35]. Transcriptional levels of *MdMYB1* are correlated with anthocyanin biosynthesis in the peel of ‘Granny Smith’ fruits [36], which display reduced methylation in the − 2026 to − 1870 bp and − 1062 to − 964 bp regions of the *MdMYB1* promoter after bag removal [11]. Expression of the structural genes *MdF3H*, *MdDFR*, *MdANS*, and *MdUFGT* that are involved in anthocyanin biosynthesis is considerably increased in ‘Granny Smith’ apple peel after bag removal [36]. However, the signal transduction pathways mediating light-induced anthocyanin accumulation in ‘Granny Smith’ after bag removal are yet to be defined.

The objective of this study was to investigate the molecular mechanisms and the metabolic pathways involved in the regulation of red pigmentation induced by bagging treatment, and to identify the key genes involved in regulating anthocyanin biosynthesis in the peel of ‘Granny Smith’ apples after bag removal. Based on transcriptome sequencing of ‘Granny Smith’ fruit peel after bag removal, we identified differentially expressed genes (DEGs) involved in light responses, hormone signal transduction pathways, and enzymatic antioxidant systems. By constructing co-expression networks, we revealed the differential regulation of genes involved in anthocyanin accumulation. The findings help to further clarify the regulatory mechanisms of bagging treatment-induced red pigmentation in ‘Granny Smith’ apples and possibly other non-red fruits.

## Results

### Changes in fruit pigmentation patterns in apple peel

**Fig. 1:**
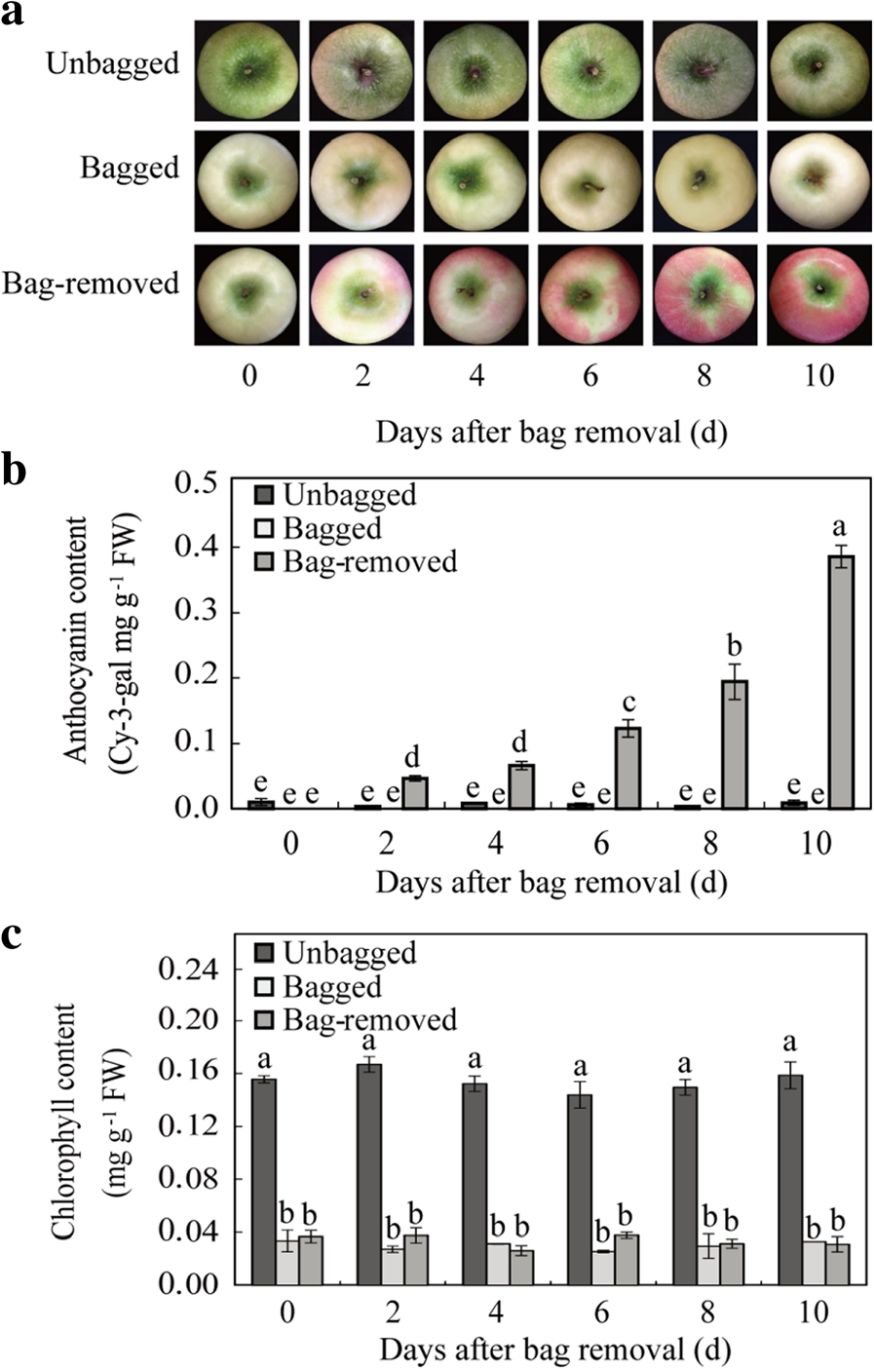
Changes in peel color and pigmentation in the ‘Granny Smith’ apple cultivar after bag removal. **a** Color development in apple peel. **b** Changes in cyanidin3-galactoside and (**c**) chlorophyll content in fruit peel. Error bars indicate standard deviation of biological replicates. Different lowercase letters indicate significant differences among bag-removed, unbagged, and bagged fruits by Tukey’s multiple range tests (*p* < 0.05)

By visual inspection, fruits gradually turned red after bag removal, while unbagged and bagged fruits remained green and white, respectively (Fig. 1a). The red pigmentation of fruits removed from bags [160 days after full bloom (DAFB)] was associated with an elevation (0 to 0.39 mg g^− 1^) in the anthocyanin content of fruit peel samples from 0 to 10 days after bag removal (DABR), while only trace amounts of anthocyanins were observed in unbagged (~ 0.01 mg g^− 1^) and bagged (0 mg g^− 1^) fruits (Fig. 1b). Additionally, the chlorophyll content in the peel of bagged fruits and bag-removed fruits remained at ~ 0.04 mg g^− 1^, which was significantly lower than in unbagged fruits; no significant difference was observed in chlorophyll content between bagged and bag-removed fruits (Fig. 1c). These findings indicate that the red pigmentation can be mainly attributed to the accumulation of anthocyanins in the peel of ‘Granny Smith’ apples.

### Characterization of unbagged, bagged and bag-removed apple transcriptomes

To gain global insight into the molecular mechanisms underpinning the different phenotypes, we analyzed fruit peel samples of unbagged (U), bagged (B), and bag-removed (R) groups at 0 (U0/B0/R0), 4 (U4/B4/R4), and 10 (U10/B10/R10) DABR by RNA sequencing (RNA-seq). After stringent quality filtering, the number of clean reads per library ranged from 24.40 to 32.42 million (M), and the rate of reads mapped to the genome was 62.56 to 73.35%, with a uniquely mapped rate of 56.44 to 65.29% (Additional file 1: Tables S1 and S2). All biological replicates showed a strong correlation (R^2^ ≥ 0.99; Additional file 1: Table S3).

### Identification of DEGs

**Fig. 2:**
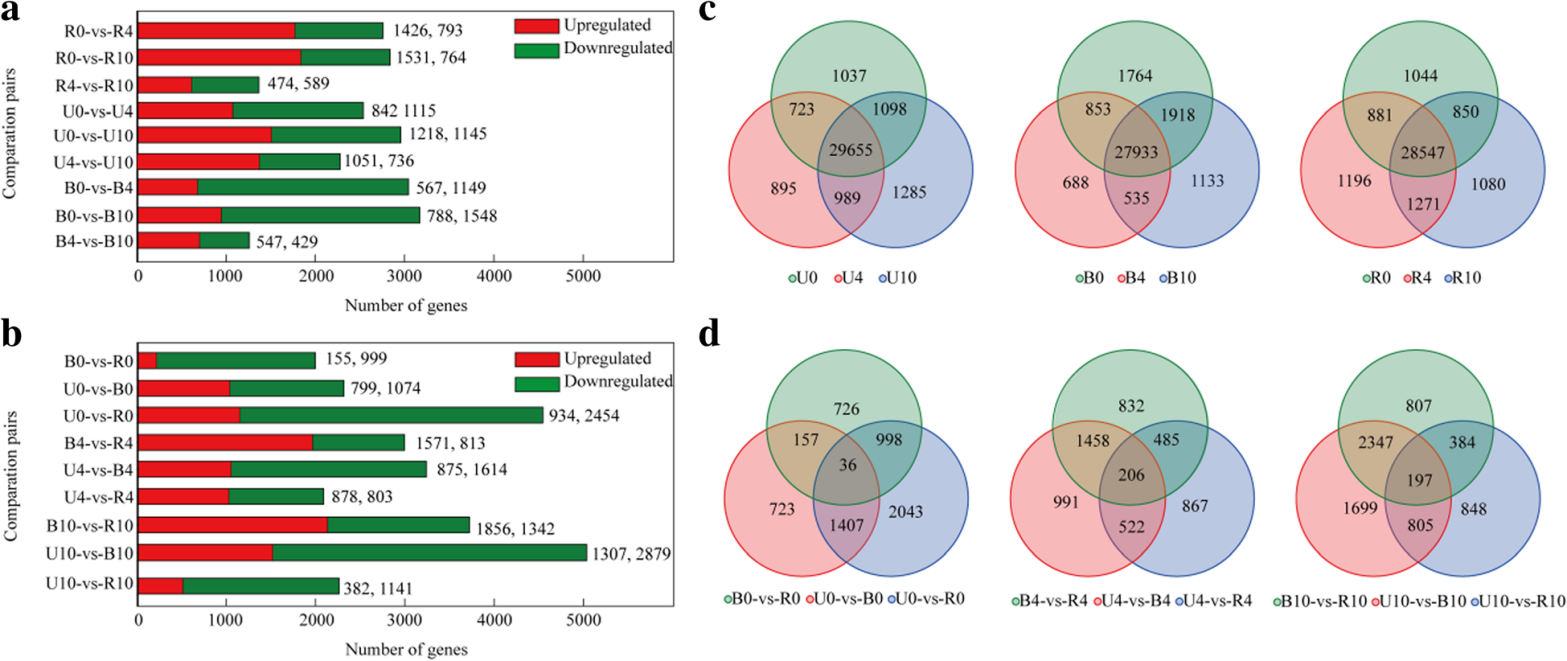
Differentially expressed genes (DEG) identified by RNA-seq analysis in the peel of bag-removed (R), unbagged (U), and bagged (B) fruits at varying developmental stages. **a** Number of DEGs between samples in each treatment groups at 0, 4, and 10 days after bag removal (DABR) (**b**) Number of DEGs between U vs. R, U vs. B, and B vs. R groups at 0, 4, and 10 DABR (**c**) Venn diagram representation of DEGs from pairwise comparisons. **d** Venn diagram representation of total genes from 0 to 10 DABR in R, U, and B groups. FDR < 0.05 and |log_2_FC| > 1 were used as cut-off criteria for significance. 0-, 0 DABR; − 4, 4 DABR; and − 10, 10 DABR

DEGs were identified among samples of each treatment group at 0, 4, and 10 DABR. More DEGs were found between R0 vs. R4 than R4 vs. R10, and similar results were also obtained for the B and U groups (Fig. 2a and c). This result indicates that the stage between 0 and 4 DABR may be the critical period affecting fruit pigmentation and development in apple peel. DEGs between and among different treatment groups were also identified. The number of DEGs between U vs. B (or B vs. R) slightly increased following bag removal, while the reverse was true for that between U vs. R. The identified DEGs were selected for further analysis (Fig. 2b and d).

### Changes in gene expression profiles in different treatment groups

**Fig. 3:**
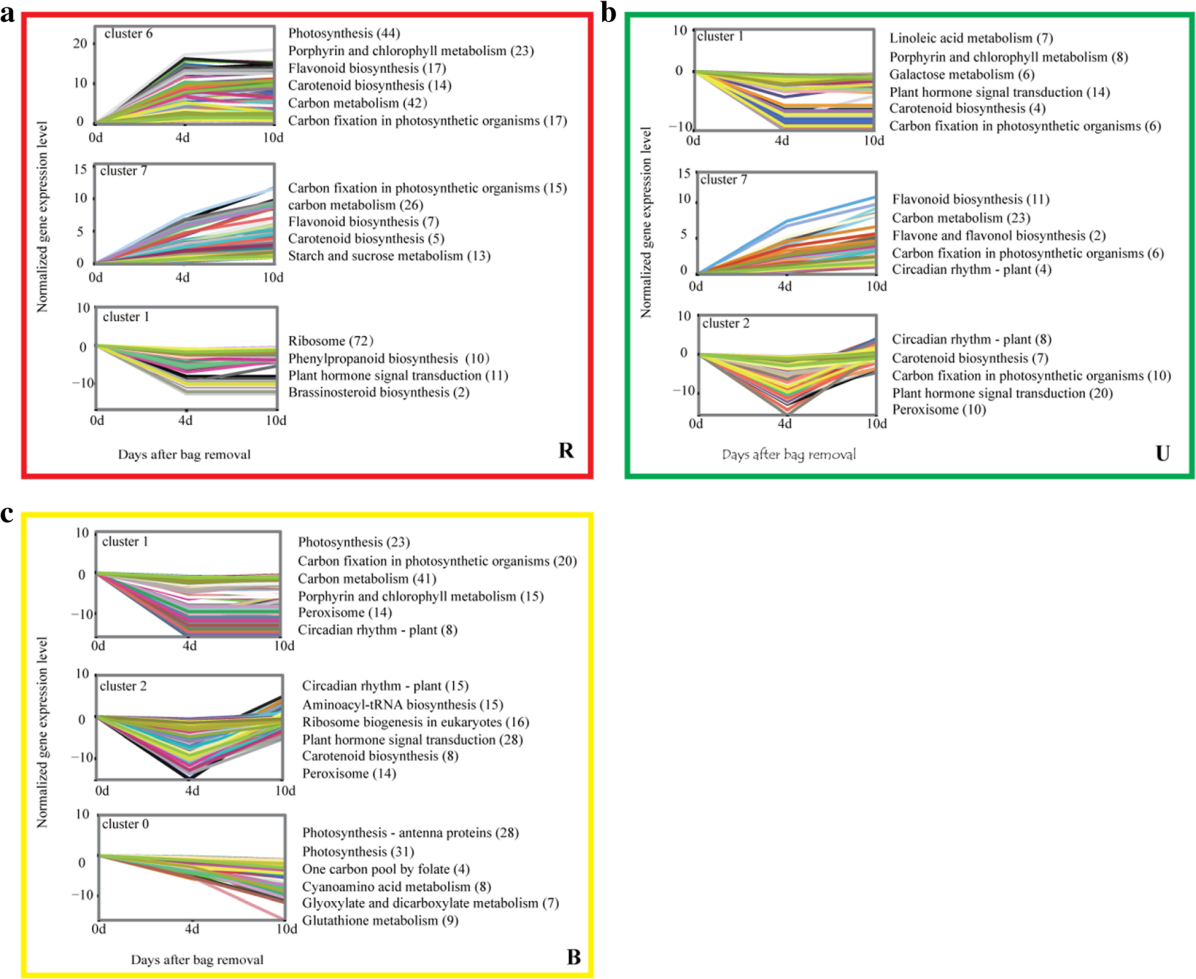
Cluster analysis and Kyoto Encyclopedia of Genes and Genomes (KEGG) pathway enrichment analysis of differentially expressed transcripts exhibiting significant expression profile changes, and KEGG pathway enrichment analysis. Enriched KEGG pathways are listed to the right of each cluster. (**a**), (**b**) and (**c**) Changes in gene expression profiles and KEGG pathways in bag-removed, unbagged, and bagged fruits, respectively

To gain further insight into related biological processes, transcripts could be divided into eight clusters in each treatment group (Additional file 2: Figure S1), representing distinct expression patterns. Then, we performed Kyoto Encyclopedia of Genes and Genomes (KEGG) pathway enrichment analysis to provide a global perspective of the biological pathways enriched in each cluster of similarly regulated transcripts (Fig. 3). Cluster 7 in both the R and U groups contained genes positively modulated throughout the entire time course, many of which were involved in flavonoid biosynthesis (Fig. 3a and b). Cluster 6 in the R group contained genes upregulated at 0 DABR, including those involved in photosynthesis, porphyrin and chlorophyll metabolism, carotenoid biosynthesis, carbon metabolism, and carbon fixation in photosynthetic organisms (Fig. 3a). Reversely, the above-mentioned genes were significantly downregulated from 0 to 4 DABR in the B group (Fig. 3c). In addition, genes involved in the ‘peroxisome’ category were significantly downregulated from 0 to 4 DABR in the U and B groups (Fig. 3b and c). These results indicate these biological pathways have potentially important effect on apple color.

### Expression of genes involved in light signal perception and transduction

**Fig. 4:**
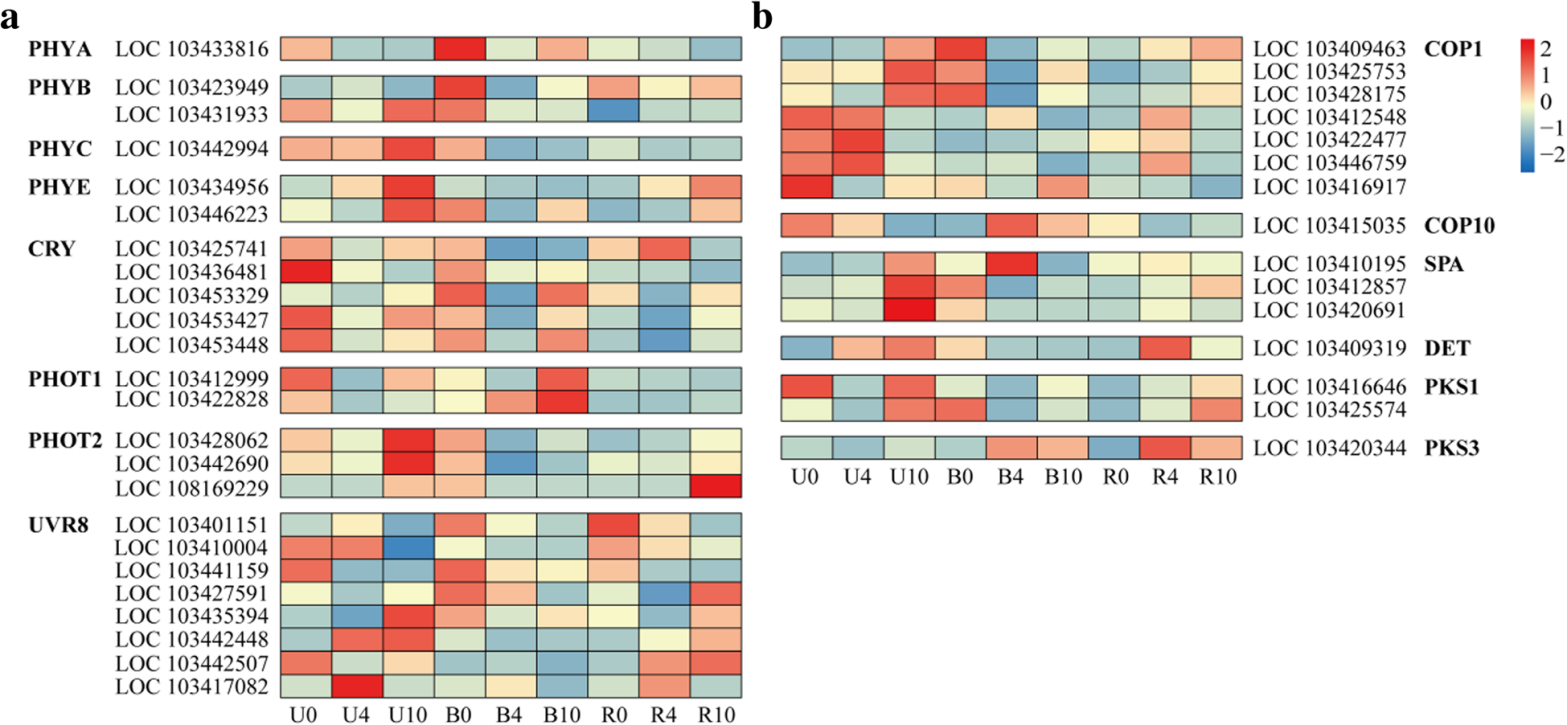
Heat map representation of the expression patterns of photoreceptors (**a**) and genes related to light signal transduction (**b**) in apple peel. PHYA, Phytochrome A; PHYB, Phytochrome B; PHYC, Phytochrome C; PHYD, Phytochrome D; PHYE, Phytochrome E; CRY, Cryptochrome; PHOT, Phototropin; UVR8, UV resistance locus 8; COP1, Constitutive hotomorphogenic 1; COP10, Constitutive hotomorphogenic 10; SPA, Suppressor of phyA; DET, DE-ETIOLATED; PKS1, Phytochrome kinase substrate 1; and PKS3, Phytochrome kinase substrate 3. Enzyme names, gene IDs, and expression patterns are indicated at the side of each row. Columns and rows represent samples collected at different time points for which bags were removed. The color scale on the right represents the log-transformed fragments per kilobase of transcript per million (FPKM) value

Following bag removal, apple responses to light were mediated by different classes of photoreceptors (Fig. 4a). Both *PHYA* and *PHYC* were expressed at low levels in the R group, with the highest levels found in B0 and U10, respectively. Transcription of two *PHYBs* also remained low in the R group; LOC103423949 expression peaked in B0, while LOC103431933 expression was highest in U10 and B0. Additionally, expression of two *PHOT1s* (LOC103412999 and LOC103422828) was low in the R group, with the highest levels found in B10. By contrast, two *PHYEs* displayed high expression in U10, and levels gradually increased in R group. Among *CRYs*, expression of LOC103425741 increased rapidly in the R group and peaked in R4, whereas the other four members exhibited low expression in this group. Among *PHOT2s*, expression of LOC108169229 increased in the R group and peaked in R10, whereas LOC103428062 and LOC103442690 exhibited low expression in this group. Moreover, eight genes encoding UVR8 proteins were identified; expression of LOC103401151, LOC103410004, and LOC103441159 decreased in the R group, while levels of the other five were increased to varying degrees.

Light signal transduction genes were also among the DEGs identified in apple peel (Fig. 4b). Low expression of *COP1s* was found in the R group compared with the U and B groups. Expression of *COP10* (LOC103415035) decreased in both U and R groups, with peak levels found in B4. *SPAs* were expressed at low levels in the R group, while expression significantly increased in the U group. *DET* (LOC103409319) expression levels were highest in U10 and R4. Expression of two *PKS1s* gradually increased in the R group; LOC103416646 was highly expressed in U0 and U10, while LOC103425574 reached its peak levels in U10 and B0. In addition, *PKS3* exhibited low expression in the U group, but levels gradually increased in the other groups and peaked in B4 and R4. These results indicate that the light signal perception and transduction patterns in apple peel could be dynamically altered by bagging treatment.

### Expression of genes associated with phytohormone signaling

**Fig. 5:**
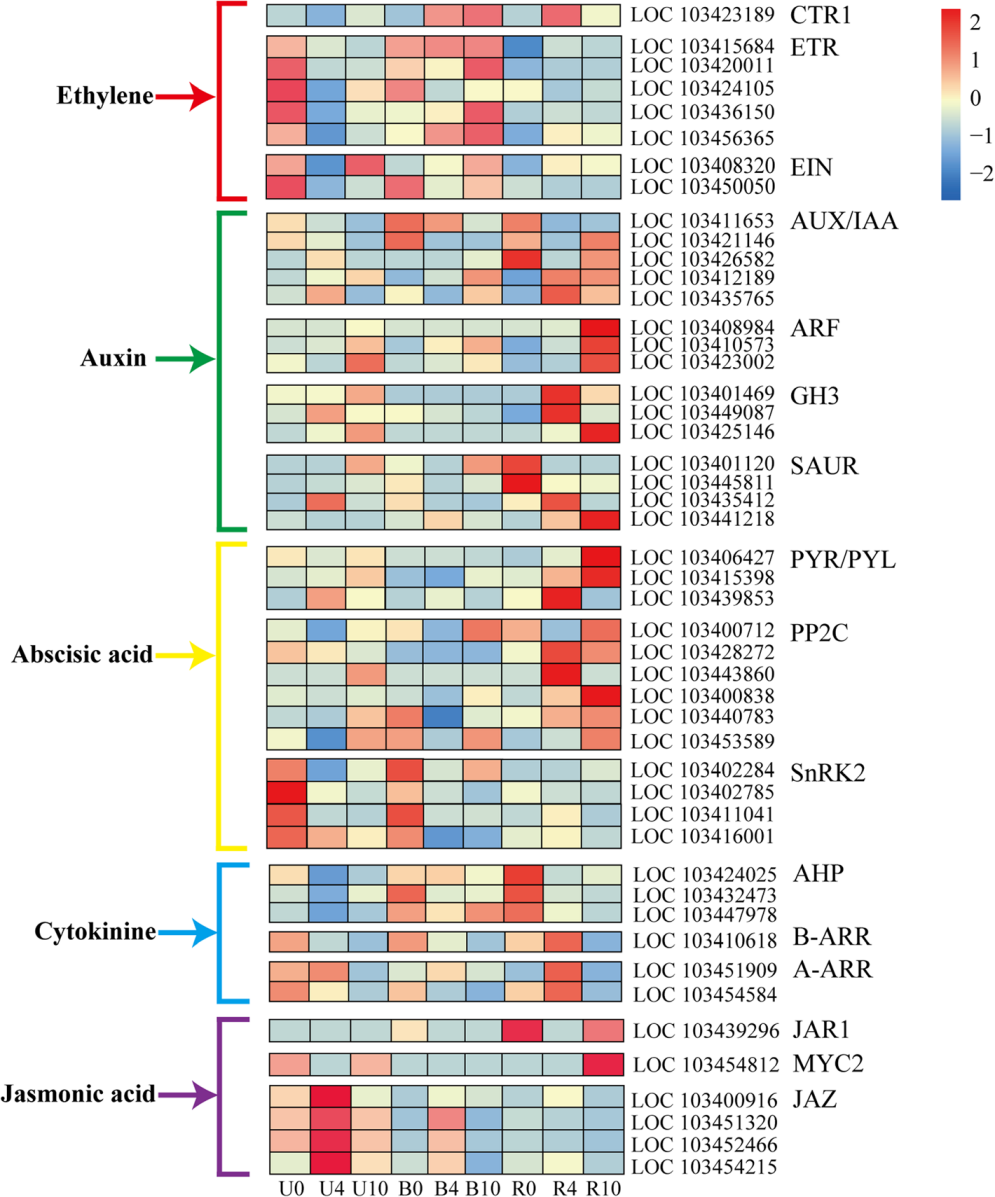
DEGs mapped to plant hormone signal transduction pathways in the KEGG database. CTR1, serine/threonine-protein kinase CTR1; ETR, ethylene receptor; EIN, ethylene-insensitive protein; AUX, auxin influx carrier; IAA, auxin-responsive protein IAA; ARF, auxin response factor; GH3, auxin responsive GH3 gene family; SAUR, small auxin up RNA; PYR/PYL, abscisic acid receptor PYR/PYL family; PP2C, protein phosphatase 2C; SnRK2, serine/threonine-protein kinase SRK2; AHP, histidine-containing phosphotransfer peotein; B-ARR, two-component response regulator ARR-B family; A-ARR, two-component response regulator ARR-A family; JAR1, jasmonic acid-amino synthetase; MYC2, transcription factor MYC2; and JAZ, jasmonate ZIM domain-containing protein. Columns and rows in the heat map represent samples collected at different time points for which bags were removed. The color scale on the right represents the log-transformed FPKM value

To determine the functions of plant hormones in bagging-induced pigmentation, we assessed the expression patterns of hormone signaling-related genes encoding receptors and response factors. The heat map shows that the functions of genes involved in plant hormone signaling differed across the three treatment groups (Fig. 5).

In the ethylene transduction pathway, the transcriptional level of serine/threonine-protein kinase CTR1 (*CTR1*, LOC103423189) was low in the U group, while this gene was upregulated in B4 and R4. Meanwhile, expression of the ethylene receptor (ETR) and ethylene-insensitive protein (EIN) genes was low in the R group. In contrast, expression levels of genes encoding auxin influx carrier/auxin-responsive protein IAA (AUX/IAA), auxin response factor (ARF), auxin responsive GH3 gene family (GH3), and small auxin up RNA (SAUR) were significantly upregulated in the R group.

In the abscisic acid transduction pathway, genes encoding the abscisic acid receptor (PYR/PYL) and protein phosphatase 2C (PP2C) were highly expressed in the R group. Reversely, expression of serine/threonine protein kinase SRK2n (*SnRK2*) genes was low in the R group, with high levels found in U0 and B0.

In the cytokinin pathway, expression of histidine-containing phosphotransfer protein (AHP) genes peaked in R0, while expression of two-component response regulator ARR-A and ARR-B families peaked in R4. Moreover, expression of the jasmonic acid-amino synthetase (JAR1) gene was high in R0 and R10, while low transcriptional levels were found in the U and B groups. Similarly, expression of the transcription factor *MYC2* peaked in R10. In contrast, expression of four jasmonate ZIM (JAZ) domain-containing genes was low in the R group, and levels peaked in U4. These results indicate that the genes involved in phytohormone signaling follow distinct expression patterns in apple peel during the bagging treatment.

### Expression of genes related to antioxidant enzyme systems

**Fig. 6:**
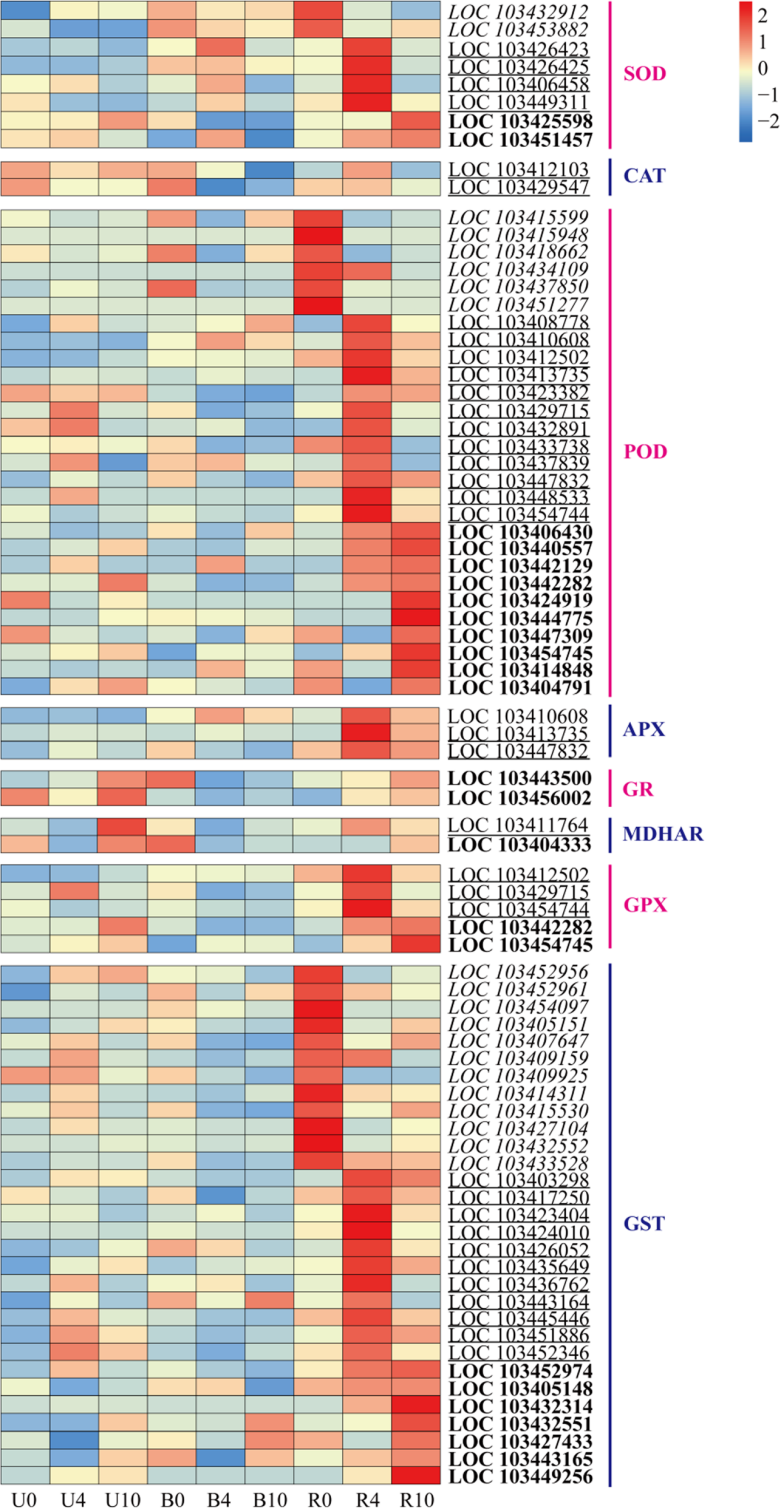
Heat map representation of the expression of genes related to antioxidant systems in apple peel. SOD, superoxide dismutase; CAT, catalase; POX, peroxidase; APX, ascorbate peroxidase; GR, glutathione reductase; MDHAR, monodehydroascorbate reductase; GPX, glutathione peroxidase; GST, glutathione S-transferase. Italicized, underlined, and bold text indicates DEGs exhibiting highest expression in R0, R4, and R10, respectively. Columns and rows in the heat map represent samples collected at different time points for which bags were removed. The color scale on the right represents the log-transformed FPKM value

To investigate the effect of light exposure on antioxidant enzyme systems, we analyzed the expression of some important antioxidant genes across the three treatment groups. *SODs*, *CATs*, *POXs*, *APXs*, *GRs*, *MDHARs*, *GPXs*, and *GSTs* were upregulated during the pigmentation of ‘Granny Smith’ fruits (Fig. 6). Notably, global gene expression patterns were higher in R than in the other two groups, except for one *CAT* (LOC103429547), two *GRs* (LOC103443500 and LOC103456002), and two *MDHARs* (LOC103404333 and LOC103411764). Following bag removal, expression of most antioxidant enzyme-encoding genes peaked in R4. However, two *SODs*, six *PODs*, and 12 *GSTs* exhibited relatively high expression in R0, followed by a decrease thereafter. These findings indicate that light exposure could induce transient and significant regulation of transcripts that encode antioxidant enzymes in apple peel. Moreover, some genes peaked in R10, including two *SODs*, 10 *PODs*, two *GPXs*, and seven *GSTs*, which exhibited similar trends as the anthocyanin content in apple peel of the R group (Fig. 1b).

### Identification of co-expression network modules associated with anthocyanin accumulation

**Fig. 7:**
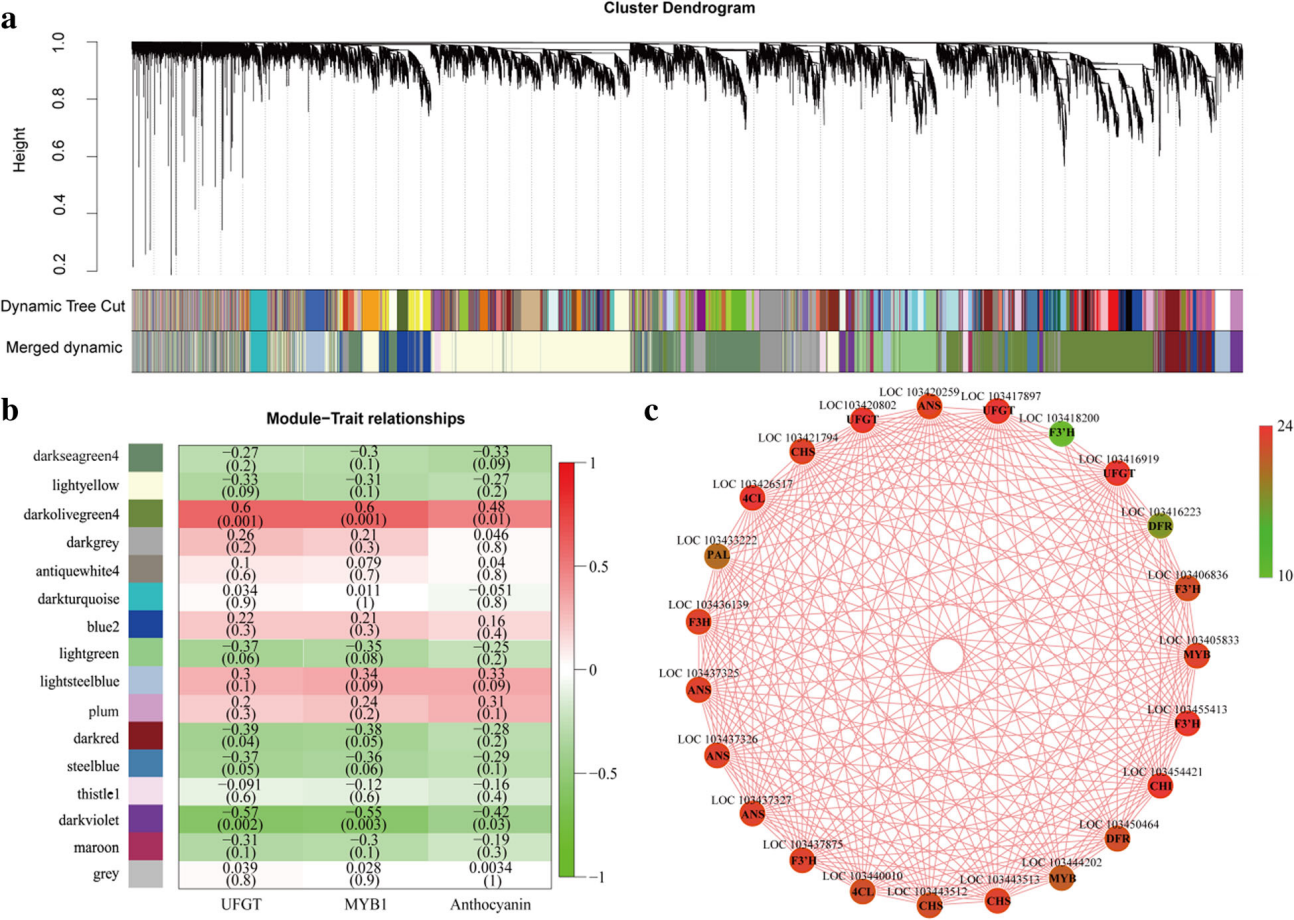
Weighted gene co-expression network analysis (WGCNA) of DEGs identified in apple peel after bag removal. **a** Hierarchical cluster tree showing 16 modules of co-expressed genes. Each of the 18,152 DEGs is represented by a leaf in the tree, and each of the modules by a major tree branch. The lower panel shows modules in designated colors. **b** Module-trait correlations and corresponding *p*-values (in parentheses). The left panel shows the 16 modules and the number of member genes. The color scale on the right shows module-trait correlations from − 1 (green) to + 1 (red). The left panel labeled ‘UFGT’ represents expression changes of *MdUFGT*, which encodes the enzyme that catalyzes the last step in anthocyanin biosynthesis, as a trait. The middle panel labeled ‘MYB1’ represents expression changes of *MdMYB1*, the key transcriptional factor activating anthocyanin biosynthesis, as a trait. The right panel labeled ‘Anthocyanin’ represents anthocyanin biosynthesis as a trait. **c** Cytoscape representation of co-expressed genes with edge weights ≥0.80 in the ‘darkolivegreen 4’ module. The edge number of genes ranges from 10 to 24 (color-coded using the scale on the right, from green to red). Member gene IDs and common names are given

A weighted gene co-expression network analysis (WGCNA) was performed on the 18,152 non-redundant DEGs identified above, leading to the identification of 16 modules (Fig. 7a and b). Analysis of module-trait relationships revealed that the ‘darkolivegreen 4’ module was highly correlated with anthocyanin content (*r* = 0.48, *p* = 0.01) in the 27 samples (Fig. 4b). Further module-trait relationship analysis, using the expression level of *MYB1* (LOC103444202) and *UFGT* (LOC103417897) as trait data, revealed that their expression patterns were indeed highly correlated with this module for the 27 samples (Fig. 7b). Therefore, genes linked to this module may play crucial roles in anthocyanin accumulation in apple peel after bag removal, and their collective repression would likely lead to loss of color in bagged fruits. In this module (‘darkolivegreen 4’), we found 22 structural genes and one *MYB1* involved in anthocyanin biosynthesis and transportation. Cytoscape representation of these 23 genes with a WGCNA edge weight ≥ 0.80 showed that they were highly connected; 21 of the 23 genes had 20 or more edges, and only two (DFR, LOC103416223; F3’H, LOC103418200) had a low number of edges (15 and 8, respectively; Fig. 7c).

### Expression of genes involved in anthocyanin biosynthesis

**Fig. 8:**
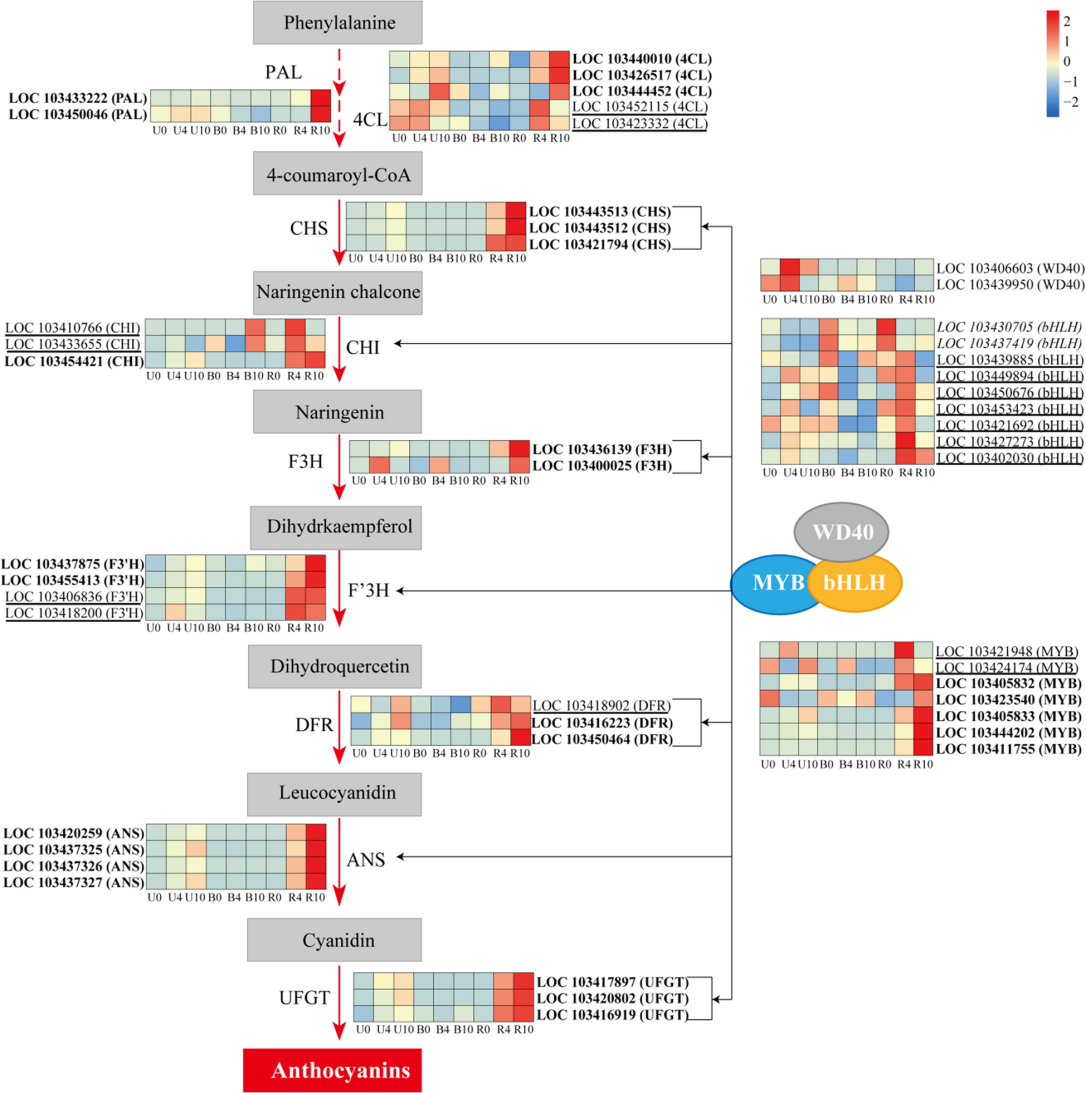
Simplified scheme and heat map of the expression of genes related to anthocyanin biosynthesis in apple peel. PAL, phenylalanine ammonia lyase; 4CL, 4-coumarate coenzyme A ligase; CHS, chalcone synthase; CHI, chalcone isomerase; F3H, flavanone 3-hydroxylase; F3’H, flavanone 3′-hydroxylase; DFR, dihydroflavonol-4-reductase; ANS, anthocyanidin synthase/leucoanthocyanidin dioxygenase; and UFGT, UDP-glucose: flavonoid-3-O-glucosyltransferase. Columns and rows in the heat map represent samples collected at different time points for which bags were removed. The color scale on the right represents the log-transformed FPKM value

In total, we identified 48 transcripts of genes potentially participating in various steps of anthocyanin metabolic pathways (Fig. 8). In the earlier stages of the anthocyanin biosynthesis pathway, transcriptional levels of the structural genes *PAL*, *4CL*, *CHS*, *CHI*, *F3H*, and *F3’H* were significantly upregulated in the R group. Expression of the two *PALs* was low in the U and B groups, while levels gradually increased in the R group. Three *4CL* genes exhibited highest levels in R10, and the other two peaked in R4. Transcriptional levels of three *CHSs* peaked in R10, and two *CHIs* increased gradually and peaked in R4, while LOC103454421 peaked in R10. Two *F3Hs* and two *F3’Hs* peaked in R10, while transcriptional levels of another two *F3’Hs* were highest in R4.

The later stages of the anthocyanin biosynthesis pathway mainly involved *DFR*, *ANS*, and *UFGT* (Fig. 8). Among *DFRs*, LOC103416223 and LOC103450464 were highly expressed and peaked in R10, while the highest expression of LOC103418902 occurred in R4. *ANSs*, *UFGTs*, and five *MYB1s* displayed similar expression patterns; they were highly expressed in the R group and levels peaked in R10. The other two *MYB1s* and seven *bHLHs* reached their highest expression levels in R4. Expression of two *bHLHs* peaked in R0. Two *WD40s* displayed high expression levels in U4, but with low expression in the R group. These data indicate most of the genes involved in anthocyanin biosynthesis play positive roles in the red pigmentation phenotype of ‘Granny Smith’ after bag removal.

### Function of the *MdMYB1* promoter in transgenic tobacco and apple

**Fig. 9:**
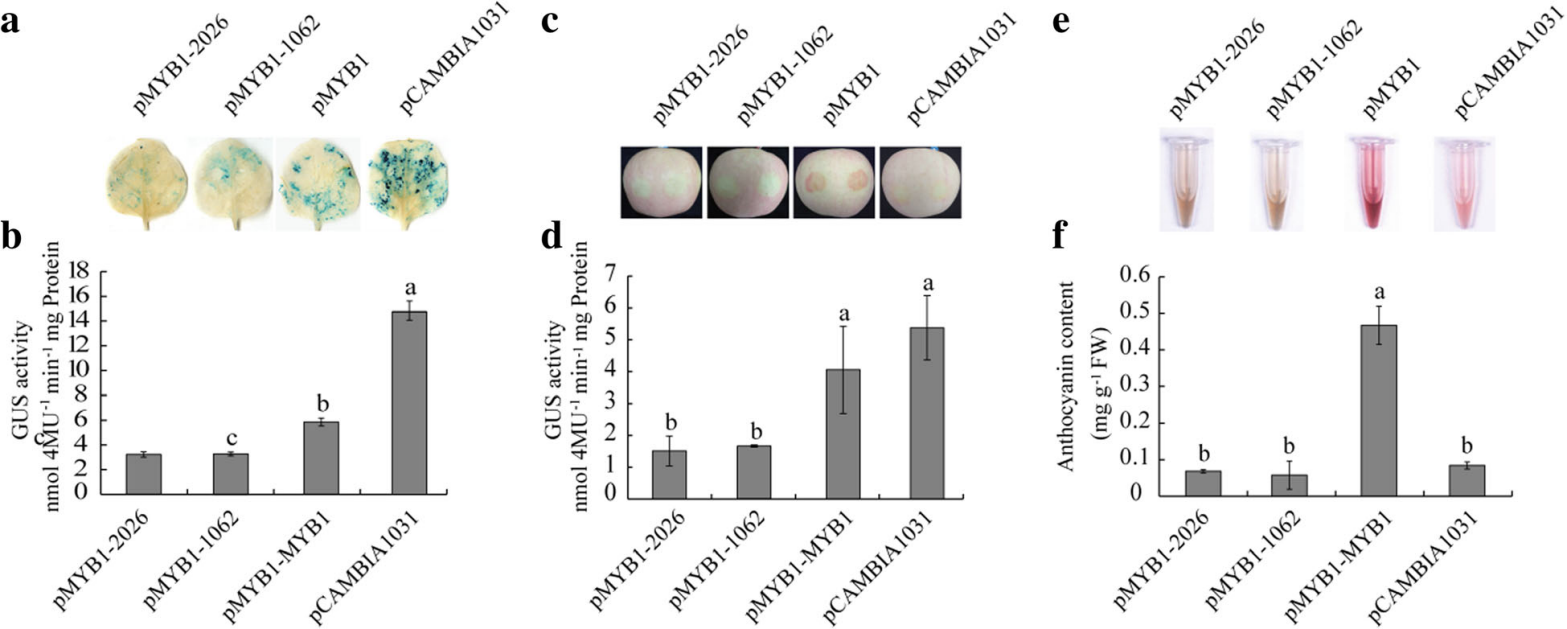
Transcriptional gene silencing of the *MdMYB1* gene in tobacco leaves and apple peel. **a** Glucuronidase (GUS) staining of infiltrated tobacco leaves. **b** GUS activity of infiltrated tobacco leaves. **c** GUS staining of the peel of infiltrated fruits. **d** GUS activity of the peel of infiltrated fruits. **e** Anthocyanins extracted from the peel of infiltrated fruits. **f** Concentration of anthocyanins in the peel of infiltrated fruits. All tests were performed in triplicate. pMYB1–2026, infiltrated with pMYB1–2026 (*MdMYB1* promoter lacking the − 2026 to − 1870 bp region); pCAMBIA1031–1062, infiltrated with pMYB1–1062 (*MdMYB1* promoter lacking the − 1062 to − 964 bp region); pMYB1, infiltrated with pMYB1 (carrying the complete sequence of the *MdMYB1* promoter); pCAMBIA1031, infiltrated with pCAMBIA103 (empty vector). Different lowercase letters indicate significant differences by Tukey’s multiple range tests (*p* < 0.05)

To test the function of the *MdMYB1* promoter, three promoter fragments (2026 bp, complete sequence; 1869 bp, lacking the − 2026 to − 1870 bp region; and 1927 bp, lacking the − 1062 to − 964 bp region) were transcriptionally fused to the promoterless reporter gene of GUS and each construct was introduced into tobacco leaves and apple peel. Compared with the complete promoter (pMYB1), GUS activity with pMYB1–2026 (*MdMYB1* promoter lacking the − 2026 to − 1870 bp region) was decreased by 44.9 and 62.6% in tobacco leaves and apple peel, respectively; with pMYB1–1062 (*MdMYB1* promoter lacking the − 1062 to − 964 bp region), the decrease was 43.3% in tobacco leaves and 59.2% in apple peel (Fig. 9b and d). The highest levels of GUS activity in tobacco leaves and apple peel were obtained with the pCAMBIA1031 (empty plasmid) construct. Additionally, fruits infiltrated with pMYB1 displayed enhanced pigmentation and increased anthocyanin accumulation around the injection sites, while pMYB1–2026 and pMYB1–1062 inhibited both anthocyanin accumulation and apple coloration (Fig. 9c, e and f). The anthocyanin content in apple peel treated with pMYB1–2026 and pMYB1–1062 was 14.7 and 12.4%, respectively, relative to pMYB1 (100%; Fig. 9f). These results indicate that pMYB1–2026 and pMYB1–1062 are essential for the increased accumulation of anthocyanins in apple peel after bag removal.

### Quantitative real-time PCR (qRT-PCR) validation of DEGs

**Fig. 10:**
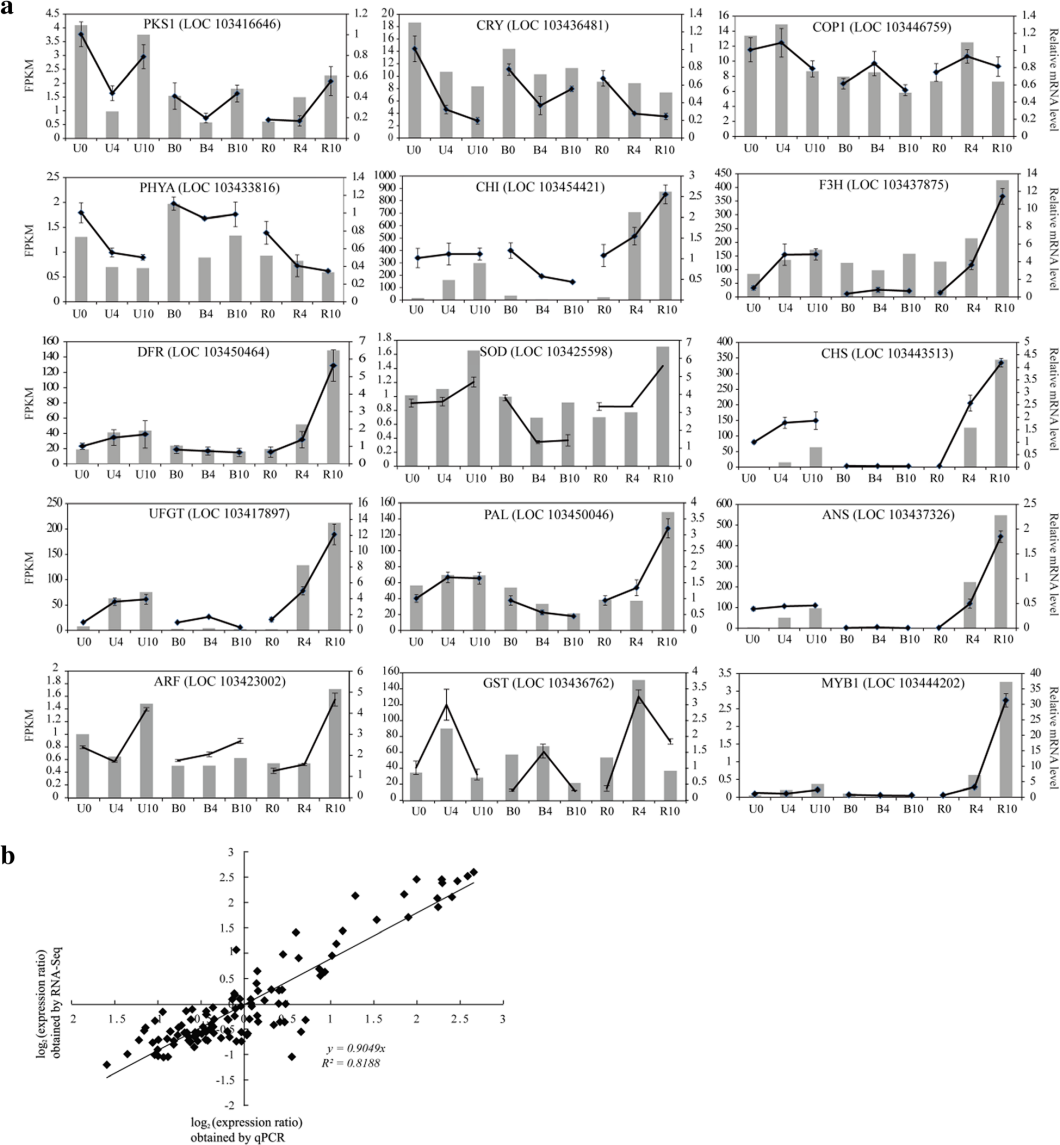
Quantitative real-time PCR (qRT-PCR) analysis of DEGs in apple peel after bag removal. **a** Transcript levels and qRT-PCR results of 15 selected genes from RNA-sequencing. The left y-axis indicates the corresponding RNA-seq data (gray histogram). The right y-axis shows the relative gene expression level measured by qRT-PCR (black lines). The x-axis represents the time (days) after bag removal. Bars represent standard error (*n* = 3). **b** Comparison between the log_2_ values of gene expression ratios obtained from RNA-seq and qRT-PCR methods

To validate the RNA-seq transcriptome results, 15 DEGs were selected for measurement of transcript levels by qRT-PCR. These genes included *CHI*, *PAL*, *F3H*, *CHS*, *DFR*, *UFGT*, and *ANS* involved in anthocyanin biosynthesis; *MYB1* that regulates anthocyanin biosynthesis; *PKS1*, *CRY*, *COP1*, and *PHYA* that are associated with photoreception and light signal transduction; *ARF* that is involved in the auxin transduction pathway; and *SOD* and *GST* antioxidant enzymes. The exact fold change of DEGs at several data points varied between RNA-seq and qRT-PCR methods (Fig. 10a). However, the overall expression patterns were strongly consistent (Pearson correlation coefficient *R*^2^ = 0.8188; Fig. 10b), confirming the reliability of the RNA-seq results.

## Discussion

### Light-induced anthocyanin accumulation is mediated by signal transduction pathways in ‘Granny Smith’

Light plays vital roles in regulating anthocyanin biosynthesis, especially in fruit peel [8, 37]. In the current study, we observed red pigmentation in the green-skinned ‘Granny Smith’ apple cultivar within a short time after bagging treatment, and anthocyanins were rapidly synthesized following bag removal (Fig. 1b). By contrast, natural and shaded conditions did not confer red pigmentation in ‘Granny Smith’ (Fig. 1a). This result indicates that bagging-induced coloration of non-red apples is mainly mediated by light, similar to observations in previous studies on lychee (*Litchi chinensis*) [15] and pear (*Pyrus pyrifolia*) [38]. Moreover, KEGG analysis showed that the genes involved in flavonoid biosynthesis were upregulated in the bag-removed group throughout the entire time course, further indicating the major effect of light on apple color.

Plants utilize multiple sensory photoreceptors to coordinate their responses to ambient light [39]. When dark-grown apple fruits were re-exposed to sunlight, we found two *PHYE*s, one *PHOT2*, and four *UVB*8s that were markedly upregulated in apple peel, with levels peaking in the bag-removed group at 10 DABR (R10; Fig. 4a). These gene expression patterns are consistent with the trend of anthocyanin accumulation we observed in apple peel (Fig. 1b). We suspect that *PHYEs*, *PHOT2*, and *UVB8*s participate in the regulation of anthocyanin biosynthesis in the peel of ‘Granny Smith’ apples. Among these photoreceptors, *UVR8s* may be the most important photoreceptor in the pericarp of lychee [15].

*COP1*s are required for the ubiquitination and degradation of the MdMYB1 protein in the dark [17], which is a positive regulator for fruit coloration [8]. In the present study, we found that expression of *COP1s* was generally low in the bag-removed group, although six *COP1s* were upregulated in this group; this result reflect the differential expression of *COP1* in response to light in ‘Granny Smith’ apples. In addition, upregulation of one *DET*, two *PKS1*s, and one *PKS3* in the bag-removed group (Fig. 4b) reflects their possible involvement in the regulation of anthocyanin accumulation in ‘Granny Smith’ apple fruits after bag removal. These results indicate that the bag-removed fruits perceive and transduce light signals to regulate anthocyanin accumulation in ‘Granny Smith’ after bag removal.

### Plant hormones are involved in bagging-induced apple coloration

The plant hormone signaling pathways can be affected by light [16, 38]. ETR is a receptor of the plant hormone ethylene. Here we found that expression of *ETRs* was lower in apple peels of the bag-removed group compared with unbagged and bagged groups (Fig. 5). Our result is consistent with ethylene suppression of anthocyanin accumulation via binding to *ETRs* in *Arabidopsis* [40] and tobacco (*Nicotiana tabacum*) [41]. CTR and EIN function in a wide range of ethylene responses in plants [42, 43]. In the present study, increased CTR expression and decreased EIN expression were found in the bag-removed group (Fig. 5). These distinct expression patterns indicate that ethylene signal has different functions in the regulation of anthocyanin biosynthesis in apple peel through various transduction pathways.

Endogenous application of auxins can retard anthocyanin accumulation in grape (*Vitis vinifera*) [44]. However, we observed significant upregulation of genes encoding auxin-responsive elements, such as *AUX/IAAs*, *ARFs*, *GH3s* and *SAURs* in the bag-removed group (Fig. 5), which implies that auxins in vivo play positive roles in bagging-induced anthocyanin biosynthesis in apple peel. In addition, abscisic acid treatment can increase anthocyanin accumulation in cherry (*Prunus avium* L.) [45], grape (*Vitis vinifera*) [46], and strawberry (*Fragaria ananassa* Duch.) [47]. By contrast, abscisic acid also has a repressive effect on anthocyanin biosynthesis in *Arabidopsis* [48]. In the present work, we observed increased transcriptional levels of several *PYRs/PYLs* and *PP2Cs*, and low expression of *SnRK2s* in the bag-removed group (Fig. 5). This result indicates that abscisic acid is involved in bagging-induced anthocyanin biosynthesis via altering expression patterns of particular genes.

Cytokinins are positive regulators of the anthocyanin biosynthesis pathway and they function by the modulation of *PAL*, *CHI*, *CHS*, and *DFR* expression in *Arabidopsis* [49]. Cytokinin signaling is mediated by *AHPs*, *B-ARRs*, and *A-ARRs* in *Arabidopsis* [50]. Herein, we found that expression of most *AHPs* was decreased in the bag-removed group, in contrast to the upregulation of *B-ARR* and two *A-ARRs* in this group (Fig. 5). This result allows us to postulate that cytokinins are part of a complicated system regulating anthocyanin accumulation in apple peel. Furthermore, JAZ proteins directly interact with *bHLHs* and *MYBs* in the MBW complex [51]. However, we found four *JAZs* that were downregulated in the bag-removed group (Fig. 5), indicating an increase in JAZ protein does not affect the expression or function of essential bagging-inducible anthocyanin-related *MYBs* in apple peel [38]. Taken together, our results indicate that plant hormones play crucial roles in the regulation of fruit coloration in ‘Granny Smith’ after bag removal. In plants, light-dependent regulation of anthocyanin formation can be regulated via the interactions among different plant hormones [25]. Exactly how these hormones are involved in fine-tuning the regulation of light-induced anthocyanin biosynthesis and the interactions among them remain to be elucidated.

### ROS scavenging enzymes in apple peel are altered after bag removal

Production of ROS in plants causes oxidative stress [52], and various photoprotective mechanisms in red and non-red apple peel operate under high light conditions [33]. In this study, differences in the expression of antioxidant enzyme genes were evident across the three treatment groups, reflecting the dynamic responses of apple peel to sunlight exposure.

SOD provides the first line of defense against the toxic effects of elevated ROS levels [32]. Here we found, that *SODs* were upregulated in the bag-removed group (Fig. 6), indicating their involvement in combating oxidative stress in apple peel in response to sunlight exposure. Although transcription of *CATs* was upregulated in the bag-removed group, one *CAT* gene was highly expressed in the bagged group (Fig. 6). We suspect that CAT isozymes may be regulated temporally and spatially, and may respond differentially to light in apple [53, 54]. Moreover, expression of *APXs* and *PODs* was increased when dark-grown apples were exposed to light (Fig. 6). APX may increase POD activity, thereby enhancing the ROS scavenging system and improving the oxidative stress tolerance [55].

Interestingly, expression of one *GR* and one *MDHAR* was higher in the unbagged group at 10 DABR (U10) and bagged group at 0 DABR (B0), compared with the bag-removed group, in response to long-term sunlight exposure and long-term darkness, respectively (Fig. 6). Thus, these proteins may play crucial roles in plant responses to various stresses [56, 57]. Overexpression of *GPX* enhances plant tolerance to various abiotic stresses [58, 59]. In the present study, significantly increased expression of *GPXs* was observed in the bag-removed group (Fig. 6), indicating their possible involvement in responding to the transition from long-term darkness to light exposure in apple. Additionally, expression levels of *GSTs* were increased in the bag-removed group than the other two groups (Fig. 6), which is consistent with the previous finding of GST family members mediating anthocyanin transport in ‘Red Bartlett’ pears (*Pyrus communis* L.) [60]. Thus, when dark-grown apple fruits were re-exposed to sunlight, transcriptional levels of some antioxidant enzyme genes increased (Fig. 6), with their levels being correlated with anthocyanin accumulation in fruit peel (Fig. 1b). We speculate that the higher expression of antioxidant enzyme genes is involved in anthocyanin accumulation in ‘Granny Smith’ after bag removal. However, the mechanisms of the enzymatic antioxidant systems responsible for anthocyanin biosynthesis in apple peel following bagging treatment require further analysis.

### A WGCNA module is strongly associated with anthocyanins, and its implications

The transcriptomes of unbagged (green peel), bagged (white peel), and bag-removed (red peel) groups were characterized to identify DEGs, and thereby gain insight into the molecular mechanisms underpinning the regulation of anthocyanin biosynthesis and accumulation in apple peel. The identified DEGs likely represent the genes most closely associated with anthocyanins because they not only include DEGs among the treatment groups at three developmental stages, but also cover DEGs between two adjacent stages within each treatment group. The results obtained thus account for color progression (in bag-removed group) that may not be captured by direct comparison of unbagged, bagged, and bag-removed groups. The WGCNA network of DEGs revealed the ‘darkolivegreen 4’ module containing 23 genes highly correlated with anthocyanin content (Fig. 7); thus upregulation of genes in this module may result in red pigmentation in apple fruits after bag removal. Examining these 23 genes in the three treatment groups demonstrated that their expression was significantly correlated with anthocyanin content in apple peel following bag removal (Figs. 8 and 1b); this indicates these genes may play critical roles in red pigmentation in the ‘Granny Smith’ cultivar after bagging treatment.

Furthermore, we identified other structural and transcription factor-encoding genes in apple peel. Notably, structural genes generally exhibited a similar expression pattern to those of *MYBs* in apple fruits after bag removal (Fig. 8). Our result further indicats that *MdMYB* plays an positive role in anthocyanin accumulation in apple peel following bag removal [34, 36]. The − 2026 to − 1870 bp and − 1062 to − 964 bp regions of the *MdMYB1* promoter comprise MYB-binding sites and light-responsive elements. Hypomethylation of these two regions is associated with pigmentation in ‘Granny Smith’ apples after bag removal [11]. Here deletion of these two regions of the *MdMYB1* promoter induced a significant decrease in GUS activity and anthocyanin accumulation (Fig. 9); thus, these regions are essential for *MdMYB1*-mediated regulation of anthocyanin accumulation and pigmentation in the ‘Granny Smith’ cultivar. Since many genes in the WGCNA module were not characterized, this study not only provides new insight into the anthocyanin biosynthesis pathway, but also presents a list of candidate genes for more dedicated functional studies in the future.

## Conclusions

In this study, the green skinned ‘Granny Smith’ apple cultivar underwent red pigmentation after bagging treatment, while unbagged and bagged fruits did not change color. Following bag removal, expression of *PHYE*, *PHOT2*, *UVB*8, *DET*, *PKS1*, *PKS3*, and *COP1* involved in light signal perception and transduction was tightly correlated with anthocyanin biosynthesis in apple peel. Additionally, hormone signaling-related genes might be involved in bag-induced red pigmentation through differences in expression patterns. Furthermore, most antioxidant genes including *SOD*, *CAT*, *POD*, *APX*, *GPX*, and *GST* were upregulated. The red pigmentation of ‘Granny Smith’ apples is likely a consequence of the collective upregulation of 23 key genes that form the ‘darkolivegreen 4’ module in the WGCNA network. In particular, *MdMYB1* is one of the genes most upregulated in ‘Granny Smith’ after bag removal, and the − 2026 to − 1870 bp and − 1062 to − 964 bp regions of this promoter element are essential for *MdMYB1*-mediated regulation of anthocyanin accumulation and red pigmentation in the ‘Granny Smith’ apple cultivar. The transcriptome data and identified DEGs provide valuable information and candidate genes for investigating the mechanisms regulating the coloration of ‘Granny Smith’ and other non-red apple cultivars.

## Methods

### Plant materials and experimental treatments

This work investigated the mechanisms underpinning red pigmentation in ‘Granny Smith’ after bag removal, based on transcriptome sequencing. ‘Granny Smith’ cultivar fruits were collected from the Baishui Apple Experimental Station of Northwest A&F University in Yangling, Shaanxi Province, China (35°21′ N, 109°55′E, elevation = 850 m). Trees were 6 years old, grafted on the M26 rootstock (*Malus domestica*), and planted at a density of 4 × 2 m. Bagging was applied as described previously [11]. Briefly, all fruits were wrapped in a paper bag (Hongtai, Shanxi, China) at 40 DAFB, and bags were removed at 160 DAFB (bag-removed group). Fruits without bagging treatment (unbagged group) and dark-grown apple fruits (bagged group) were used for comparison. Fruit samples were randomly taken at 0, 2, 4, 6, 8, and 10 days after bag removal (DABR). Each point-in-time sample consisted of 12 fruits, and three biological replicates were harvested, with at least four fruits collected from two trees per replicate. Fruit peel was collected with a peeler, immediately frozen in liquid nitrogen and stored at − 80 °C. Peel samples from 0, 4, and 10 DABR were used for transcriptome sequencing and gene expression analysis.

For deletion analysis of the *MdMYB1* promoter, ‘Pink lady’ apple fruits were bagged at 40 DAFB, and bagged fruits were collected at 160 DAFB. Tobacco (*Nicotiana benthamiana*) plants were grown in vitro at 25 °C on Murashige-Skoog medium [61].

### Anthocyanin and chlorophyll measurement

Measurement of anthocyanin content was performed as described previously [35]. Apple peel (0.5 g) was finely ground in 5 mL HCl/methanol (1/99, v/v), and then centrifuged at 13,000×*g* for 10 min. A high-performance liquid chromatography instrument with a photodiode array detector (Waters, Milford, USA) was used for analysis. Separation of anthocyanins was accomplished on a C18 column (5 μm internal diameter, 250 × 4.6 mm; Waters). Cyanidin 3-galactoside served as a standard (Sigma-Aldrich, St. Louis, USA). Chlorophyll was extracted using a UV/visible spectrophotometer (UV-2550, Shimadzu Corp., Kyoto, Japan) as described previously [62]. Three independent biological replications were performed for each experiment.

### RNA extraction, library preparation, and RNA-seq

Total RNA was extracted from fruit peel using the TRIzol RNA plant plus reagent (Tiangen, Beijing, China) following the manufacturer’s instructions. RNA quality was assessed on an Agilent 2100 Bioanalyzer (Agilent Technologies, Palo Alto, USA). Samples with an RNA integrity number score > 7.5 were selected for deep sequencing. Next, mRNAs were enriched and cleaved into small pieces, which served as templates for cDNA synthesis. Purification of the cDNA fragments and PCR amplification were performed as described previously [35]. In total, nine sets of raw reads were obtained, corresponding to the three treatment groups at 0 (U0/B0/R0), 4 (U4/B4/R4), and 10 (U10/B10/R10) DABR. To verify the reproducibility of the sequencing data, we calculated Pearson’s correlation coefficients for three biological replicates at each developmental stage using the log_10_ fragments per kilobase of transcript per million mapped reads (FPKM) method [63].

### RNA-seq data processing and mapping of reads to the apple genome

Raw read processing of transcript datasets was carried out by Genedenovo Biotechnology Co., Ltd. (Guangzhou, China). Raw reads were further filtered according to the method of Ma et al. [35]. All clean reads were mapped to the apple (*M. domestica*) genome sequence [64] using SOA-Paligner/soap2 [65]. Clean reads were then aligned with the reference genome and transcripts were reconstructed using Cufflinks [66].

### Identification of DEGs

All clean reads were aligned to genes using TopHat2 [67] and then normalized into FPKM reads [63]. DEGs among different treatment groups at each of the three developmental stages were identified, along with DEGs between adjacent stages within each group. The original *p*-values were adjusted using the Benjamini and Hochberg approach [68] to minimize the false discovery rate (FDR). DEGs with |fold change| ≥2 and FDR < 0.05 were considered significant.

### Functional annotation

For Gene Ontology (GO) term enrichment analysis, all DEGs were mapped to GO terms in the GO database (http://www.geneontology.org/) [69]. KEGG pathway enrichment analysis was performed using the KEGG database (http://www.genome.jp/kegg/) [70]. Significant GO/KEGG enrichment compared with the genomic background was determined using hypergeometric tests. Calculated *p*-values were subjected to FDR correction, and FDR ≤0.05 was applied as the threshold.

### Construction of co-expression networks

Co-expression network analysis was performed using an R package for WGCNA [71]. WGCNA network construction and module detection was conducted using an unsigned type of topological overlap matrix, a power β of 10, a minimal module size of 50, and a branch merge cut height of 0.7. The module eigengene (the first principal component of a given module) value was calculated and used for evaluation of the association between modules and anthocyanin content in the 27 samples. The most significant module (‘darkolivegreen 4’) based on 23 genes with a WGCNA edge weight > 0.80 was represented using Cytoscape 3.3.0 [72]. Expression data were clustered using Cluster 3.0 [18] and displayed using Java Treeview [73].

### Vector construction and transformation in tobacco and apple

To analyze the function of the *MdMYB1* promoter, a 2026 bp promoter fragment (the complete sequence), a 1869 bp fragment (*MdMYB1* promoter lacking the − 2026 to − 1870 bp region), and a 1927 bp fragment (*MdMYB1* promoter lacking the − 1062 to − 964 bp region) were cloned from genomic DNA extracted as described previously [11]. Overlap extension PCR was used according to Wurch et al. [74]. Specific PCR primer information is listed in Additional file 2: Tables S4 and S5. After digestion with restriction enzymes *Xba*I and *Nco*I, the three promoter fragments were subcloned into the binary plasmid pCAMBIA1031 to replace the *CaMV35S* promoter, resulting in a series of pMYB1-del promoter::glucuronidase (GUS) fusion expression vectors, namely pMYB1–2026 (*MdMYB1* promoter lacking the − 2026 to − 1870 bp region), pMYB1–1062 (*MdMYB1* promoter lacking the − 1062 to − 964 bp region), and pMYB1 (complete sequence of the *MdMYB1* promoter). The empty pCAMBIA1031 plasmid served as a control.

Inoculation of tobacco leaves and apple peel with *Agrobacterium tumefaciens* strain EHA105 cells was conducted as previously described [17, 75]. Four expression vectors, pCAMBIA1031, pMYB1–2026, pMYB1–1062, and pMYB1, were injected into tobacco leaves and bagged apple fruit peel using a needleless syringe. Injected samples were incubated overnight in the dark at room temperature, then exposed to white light (540 μmol·m^− 2^·s^− 1^) with a 16 h photoperiod at 25 °C in a growth chamber. After 2 weeks, the anthocyanin content in injected apples was determined using a UV-2550 UV/visible spectrophotometer as previously described [76].

### Histochemical and fluorimetric GUS activity analysis

For histochemical staining of GUS, excised tobacco leaf and apple peel discs were immediately treated with 1 mM 5-bromo-4-chloro-3-indolyl-b-D-glucuronide in 100 mM phosphate buffer pH 7.0, 10 mM EDTA, 0.5 mM potassium ferrocyanide, and 0.1% Triton X-100 [77] at 37 °C for 24 h. Stained samples were bleached with 70% (v/v) ethanol, and GUS activity was determined by measuring the fluorescence of 4-methylumbelliferone produced by GUS cleavage of 4-methylumbelliferyl-β-D-glucuronide (Sigma) as described previously [78]. The protein concentration in the supernatant was determined using the Bradford procedure with bovine serum albumin (Sigma) as a standard. Three independent biological replications were performed for each experiment.

### qRT-PCR analysis

qRT-PCR analysis was performed according to Ma et al. [35]. Actin (GenBank: GQ339778.1) was used as a reference gene. Specific primers were designed using Primer 5 (Additional file 2: Table S6). Data were analyzed using the 2^-∆∆CT^ method [79].

### Statistical analysis

All data were subjected to a one-way analysis of variance. Significant differences between group means were assessed by Tukey’s post hoc tests (*p* < 0.05) using SPSS 16.0 Statistics (SPSS Inc., Chicago, IL, USA).

## Additional files

Additional file 1: **Table S1.** Statistics related to the quality and output of RNA-seq libraries. **Table S2.** Throughput and quality of RNA-seq of differentially expressed genes libraries from “Granny Smith” apple peels. **Table S3.** Pearson’s correlation coefficients of RNA-seq data for three biological replicates at each developmental stage. (XLSX 19 kb)

Additional file 2: **Table S4.** DNA sequences of oligonucleotide primers used in the *MdMYB1* promoter sequence analysis. **Table S5.** DNA sequences of oligonucleotide primers used in the overlap extension PCR. **Table S6.** Primer sequences used in the quantitative real-time PCR. **Figure S1.** Clusters of differentially expressed transcripts with expression profile changes based on the number of genes and the significance of expression profiles. (DOCX 80 kb)

## Abbreviations

4CL: 4-coumarate coenzyme A ligase
A-ARR: Two-component response regulator ARR-A family
AHP: Histidine-containing phosphotransfer protein
ANS: Anthocyanidin synthase/leucoanthocyanidin dioxygenase
APX: Ascorbate peroxidase
ARF: Auxin response factor
AUX/IAA: Auxin influx carrier/auxin-responsive protein IAA
B: Bagged
B0/4/10: Bagged fruits at 0, 4, 10 DABR, respectively
B-ARR: Two-component response regulator ARR-B family
CAT: Catalase
CHI: Chalcone isomerase
CHS: Chalcone synthase
COP1: CONSTITUTIVE PHOTOMORPHOGENIC 1
CRY: Cryptochrome
DABR: Days after bag removal
DAFB: Days after full bloom
DEG: Differentially expressed gene
DET: DE-ETIOLATED
DFR: Dihydroflavonol 4-reductase
F3H: Flavanone 3-hydroxylase
GH3: Auxin responsive GH3 gene family
GO: Gene Ontology
GPX: Glutathione peroxidase
GR: Glutathione reductase
GST: Glutathione-S-transferase
HY5: LONG HYPOCOTYL 5
JAR1: Jasmonic acid-amino synthetase
JAZ: Jasmonate ZIM domain-containing protein
KEGG: Kyoto Encyclopedia of Genes and Genomes
MBW: MYB-bHLH-WD40/WDR
MDHAR: Monodehydroascorbate reductase
MYC2: Transcription factor MYC2
PAL: Phenylalanine ammonia lyase
PHOT: Phototropin
PHY: Phytochrome
PKS1: PHYTOCHROME KINASE SUBSTRATE 1
pMYB1: Complete sequence of the *MdMYB1* promoter
pMYB1–1062: *MdMYB1* promoter lacking the − 1062 to − 964 bp region
pMYB1–2026: *MdMYB1* promoter lacking the − 2026 to − 1870 bp region
POD: Peroxidase
PP2C: Protein phosphatase 2C
PYR/PYL: Abscisic acid receptor
qRT-PCR: Quantitative real-time
R: Bag-removed
R0/4/10: Bag-removed fruits at 0, 4 and 10 DABR, respectively
ROS: Reactive oxygen species
SAUR: Small auxin up RNA
SnRK2: Serine/threonine protein kinase SRK2n
SOD: Superoxide dismutase
SPA: Suppressor of phyA
U: Unbagged
U0/4/10: Unbagged fruits at 0, 4, 10 DABR, respectively
UFGT: UDP-glucose:flavonoid 3-O-glucosyltransferase
WGCNA: Weighted gene co-expression network analysis
