# Supplementary material for: Transcriptome profiling of anthocyanin biosynthesis in the peel of ‘Granny Smith’ apples (Malus domestica) after bag removal
Source: BMC Genomics. 2019 May 9;20:353. doi: 10.1186/s12864-019-5730-1 (PMC6507055; doi:10.1186/s12864-019-5730-1)

**Table S4.** DNA sequences of oligonucleotide primers used in the *MdMYB1* promoter sequence analysis.

| Primer position | Name | Sense primer (5ʹ–3ʹ) | Anti-sense primer (5ʹ–3ʹ) |
| --- | --- | --- | --- |
| **-**2026 | *MdMYB1* | CTCTAGAGAAATCGTTCGAAGGTC | CCCATGGCTCTTATCTGCTAGC |
| -1870, -1 | *MdMYB1*-a | CTCTAGATGCCATTTTTGAACACA | CCCATGGCTCTTATCTGC |
| -964, -1 | *MdMYB1*-b1 | CTCTAGAGAAATCGTTCGAAGGTC | TCAGGCTTGTCCTACTTGGTTATCT |
| **-**2026, -1062 | *MdMYB1*-b2 | GTAGGACAAGCCTGAAATCTTAGGA | CCCATGGCTCTTATCTGCTAGC |

**Table S5.** DNA sequences of oligonucleotide primers used in the overlap extension PCR.

| Recombinant promoter | Length | Name | Primer (5ʹ–3ʹ) |
| --- | --- | --- | --- |
| *MdMYB1*-b (b1+b2) | 1927 bp | *MdMYB1*-b1F | CTCTAGAGAAATCGTTCGAAGGTC |
|  |  | *MdMYB1*-b2R | CCCATGGCTCTTATCTGCTAGC |

**Table S6.** Primer sequences used in the quantitative real-time PCR.

| Gene ID | Name | Sense primer (5ʹ–3ʹ) | Anti-sense primer (5ʹ–3ʹ) |
| --- | --- | --- | --- |
| LOC 103447714 | ACTIN | CTGAACCCAAAGGCTAATCG | ACTGGCGTAGAGGGAAAGAA |
| LOC 103450046 | PAL | AGGAACACCGTAAAGAACA | ACATACTCCCTATCGACAAC |
| LOC 103443513 | CHS | AGTGACACCCACCTTGATAG | CTGTCGGGGAGAATGGTTTG |
| LOC 103450464 | DFR | ATTTATCTTTACGAGCATCC | CCCTATCTCCCTCAACTTCT |
| LOC 103437326 | ANS | GTTCCAAATTCCATCGTCAT | TCACCTTTTCCTTGTTCACC |
| LOC 103444202 | MYB1 | GTCGTCGTCAACAAAGAATGG | GGTCCGTGCTAAAGGAGAAT |
| LOC 103454421 | CHI | GTTACAGGTCCGTTTGAGAA | AACTTTTCAATGGCTTTGCCTTCTG |
| LOC 103417897 | UFGT | TCGTAGCCTTCCCTTTCACT | TTATCAATGCTGTTGTTGGAAAAGA |
| LOC 103437875 | F3H | CTGCTACTACGCTGACATCC | AATACCCCAGTCCTCACAAG |
| LOC 103436762 | GST | ATGGGAGTGAAAGTGC | GGAAGACAATGGGAGA |
| LOC 103423002 | ARF | GAGGTGGTTTATTATCCG | TGGTGGTGAGAAGGGT |
| LOC 103425598 | SOD | GGCTGGGTTTGGCTTGT | CAGGGATGTTAGGTTCGTTT |
| LOC 103446759 | COP1 | GAGGACATTACTGCCGTTGA | AAACTGCGAATGGACACG |
| LOC 103433816 | PHYA | TGAGGAGTCGGGTAGTTC | CTTGCCTTTCTGTATGTGA |
| LOC 103436481 | CRY | TGGTCCCAAGCATTACTA | CTACACCTCCATCCCTCT |
| LOC 103416646 | PKS1 | CAGCAGTAGCAGCACGAA | CATCCACATCAACCGAAT |

**Figure S1**. Clusters of differentially expressed transcripts with expression profile changes based on the number of genes and the significance of expression profiles. (**a**), (**b**) and (**c**) Changes in gene expression profiles in bag-removed, unbagged, and bagged treatment groups, respectively. The transcripts were divided into eight clusters in each treatment group, representing distinct expression patterns, and three clusters of each group with significant differential expression at *p*-value < 0.05.


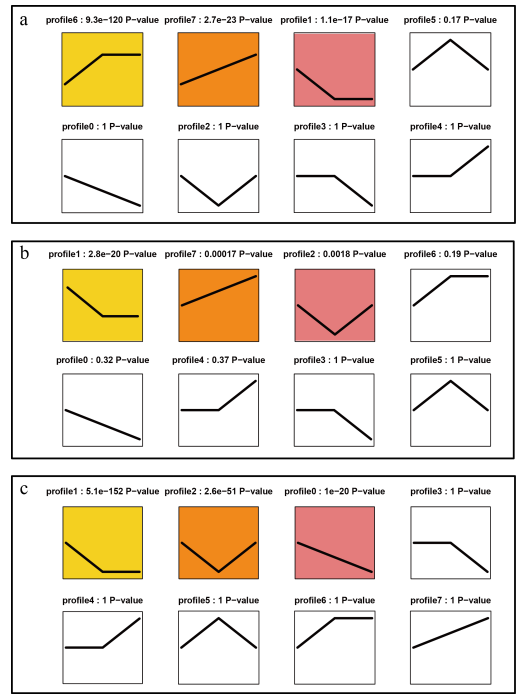

Supplement: Supplementary file 2 — Table S4. DNA sequences of oligonucleotide primers used in the MdMYB1 promoter sequence analysis. Table S5. DNA sequences of oligonucleotide primers used in the overlap extension PCR. Table S6. Primer sequences used in the quantitative real-time PCR. Figure S1. Clusters of differentially expressed transcripts with expression profile changes based on the number of genes and the significance of expression profiles. (DOCX 80 kb) [file 12864_2019_5730_MOESM2_ESM.docx]
